# Coxsackievirus A6 U.K. Genetic and Clinical Epidemiology Pre- and Post-SARS-CoV-2 Emergence

**DOI:** 10.3390/pathogens13111020

**Published:** 2024-11-20

**Authors:** Alice M. Joyce, Jack D. Hill, Theocharis Tsoleridis, Stuart Astbury, Louise Berry, Hannah C. Howson-Wells, Nancy Allen, Ben Canning, Carl B. Jones, Gemma Clark, William L. Irving, Alexander W. Tarr, C. Patrick McClure

**Affiliations:** 1 School of Life Sciences, University of Nottingham, Nottingham NG7 2RD, UK; 2 Wolfson Centre for Global Virus Research, University of Nottingham, Nottingham NG7 2UH, UK; 3 The Pirbright Institute, Ash Road, Pirbright, Woking GU24 0NF, UK; 4 Nottingham Digestive Diseases Centre, Translational Medical Sciences, School of Medicine, University of Nottingham, Nottingham NG7 2UH, UK; 5 National Institute for Health Research Nottingham Biomedical Research Centre, University of Nottingham and Nottingham University Hospitals NHS Trust, Nottingham NG7 2UH, UK; 6 Clinical Microbiology, Nottingham University Hospitals NHS Trust, Nottingham NG7 2UH, UK

**Keywords:** coxsackievirus A6, enterovirus, CVA6, genetic epidemiology, hand, foot and mouth disease, HFMD

## Abstract

Coxsackievirus A6 (CVA6) has become increasingly clinically relevant as a cause of Hand, Foot and Mouth Disease (HFMD) globally since 2008. However, most laboratories do not routinely determine the enteroviral type of positive samples. The non-pharmaceutical measures introduced to curb transmission during the COVID-19 pandemic may also have perturbed CVA6 epidemiology. We thus aimed to determine the prevalence, clinical presentation and genetic relationship of CVA6 across three complete epidemic seasons: one pre-SARS-CoV-2 emergence and two post-SARS-CoV-2 emergence in our regional healthcare setting. Surplus diagnostic nucleic acid from diagnosed enteroviral positives diagnosed between September and December of 2018 and between May 2021 and April of 2023 was subject to VP1 gene sequencing to determine the CVA6 cases and interrogate their phylogenetic relationship. The confirmed CVA6 cases were also retrospectively clinically audited. CVA6 infections were identified in 33 and 69 individuals pre- and post-pandemic, respectively, with cases peaking in November of 2018 and 2022, but in October of 2021. HFMD was the primary diagnosis in 85.5% of the post-pandemic cases, but only 69.7% of the pre-pandemic cases, where respiratory and neurological symptoms (45.5% and 12.1%, respectively) were significantly elevated. A complete VP1 sequence was retrieved for 94% of the CVA6 cases, revealing that studied infections were genetically diverse and suggestive of multiple local and international transmission chains. CVA6 presented a significant clinical burden in our regional U.K. hospital setting both pre- and post-pandemic and was subject to dynamic clinical and genetic epidemiology.

## 1. Introduction

Human enteroviral infections are contracted predominantly via the faecal–oral route and exhibit a broad spectrum of the disease, ranging from being asymptomatic or having a self-limiting illness through to experiencing high clinical consequences and the associated mortality [1,2]. The causative Enteroviruses (EV) can be subcategorised into four species, EV-A to EV-D, comprising over 100 types and are more prevalent within younger age groups, with severe outcomes including Acute Flaccid Myelitis (AFM), endocarditis and meningitis [3,4]. Less severe and more frequent enteroviral disease manifestations include hand, foot and mouth disease (HFMD), presenting classically with short-lived combinations of a low-grade fever, malaise, a maculopapular rash or blisters on the hands, soles and buttocks, and ulcerative lesions of the throat, mouth and tongue [5].

EV-A types are the leading cause of HFMD, principally EV-A71 and several members of the genetically diverse Coxsackievirus (CV) grouping CVA6, CVA10 and CVA16 [6]. Since 2008, CVA6 has been increasingly diagnosed during HFMD outbreaks across the world [7,8,9,10,11]. These recent epidemics have featured an atypical HFMD presentation of a more disseminated rash or principally herpangina [12,13,14,15] and can include a significant number of affected adults [16,17]. Onychomadesis—the separation of the nail from its matrix due to cessation in growth—can also be observed in atypical HFMD [18,19], occurring circa 8 weeks post infection in the majority of cases [20]. In rarer instances, a CVA6 infection can lead to more severe complications like aseptic meningitis, encephalitis, AFM and sometimes fatality [17,21,22,23]. Even though the majority of CVA6 infections occur without sequelae, higher mortality rates are often observed amongst the immunosuppressed due to the development of neurological complications [24]. The high prevalence of CVA6 infection combined with the potential for severity has prompted interest in the development of a vaccine [25].

HFMD is routinely diagnosed by clinical presentation; however, the causative virus can be confirmed via molecular methods involving the amplification and detection of the viral genome. Specific molecular assays targeting the 5′ UTR have previously been successful at identifying the presence of enteroviruses [26]; however, due to genetic drift occurring mainly within the capsid coding region and recombination [27,28], sequencing of the VP1 gene is used predominantly to determine EV types [29,30]. CVA6 detection can be accomplished via the analysis of skin and nasopharyngeal swabs, stool samples and/or fluid extracted from cutaneous lesions and vesicles. The European Union and the U.S.A. currently have no mandatory legislation with regards to notification of incidences of HFMD, causing difficulties with surveillance and identifying transmission trends of this enterovirus [31,32]. In contrast, HFMD is a notifiable disease in China, allowing national surveillance and enterovirus prevalence to be identified [33].

CVA6 has a single stranded positive sense RNA genome comprising approximately 7400 bases and can be distinguished into four genotypes A-D. It can be further subcategorised into sub-genotypes including B1, B2, C1, C2 and D1–3 [34]. Sub-genotype D3 has recently been evidenced as the most prevalent sub-genotype [35,36,37]; however, the non-pharmaceutical interventions during the COVID-19 pandemic may have inadvertently affected the circulation of enteroviruses including CVA6 [38]. We thus aimed to investigate the genetic and clinical epidemiology of the CVA6 cases diagnosed in our regional U.K. hospital in epidemic seasons before and after the emergence of SARS-CoV-2.

## 2. Materials and Methods

### 2.1. Clinical Sampling and Diagnosis

This study was conducted in two periods: initially between 1 September and 17 December 2018 when a significant Enterovirus D68 epidemic was experienced and previously reported [39]. A second period followed between 11 May 2021 and 26 April 2023, when our diagnostic laboratory re-commenced enteroviral testing post-pandemic and sought to evaluate routine enteroviral typing. Whilst the precise start and end of the COVID-19 pandemic are open to debate, to simplify the narrative in this study, all 2021–2023 samples are referred to as ‘post-pandemic’.

The samples were screened for enteroviral infection using both commercial and in-house assays as described previously [30,39]. Briefly, the samples were determined EV-positive by the commercial clinical assay (AusDiagnostics, Sydney, NSW, Australia) as part of the routine care diagnostic pathway. Where available, surplus nucleic acid was re-screened using in-house assays targeting the partial VP1 and 5′ NCR genomic regions and candidate positives were confirmed by Sanger sequencing [29,30]. Some patients had more than one sample taken and were thus sequenced more than once also. Additionally, a minority of the diagnostic samples were submitted for typing at the National Reference Laboratory, either confirming a CVA6 infection or independently identifying further CVA6 in 4 cases without residual material available.

### 2.2. CVA6 VP1 Gene Sequencing and Phylogenetic Investigation

Confirmed CVA6 positive samples were then further amplified with two novel PCR assays targeting overlapping genomic regions covering the entire VP1 gene, based on all CVA6 lineage exemplars as presented by Bian et al. 2015 [40] using MEGA7 [41] and Primer3 (https://primer3.ut.ee/, accessed on 1 May 2022). This pilot amplification returned inconsistent results, with approximately 50% amplification success, but Sanger sequencing and phylogenetic analysis of the successfully amplified samples indicated all were of the globally most common lineage D3 [37]. Therefore, the primers were modified and/or redesigned to specifically target CVA6 lineage D3 based on the lineage D3 reference sequences and a single contig generated from a study sample in the preliminary VP1 amplification.

The final primer pairs employed to amplify and sequence the full length VP1 were set A: CVA6D3_VP1Af AGACTGGAGGGGTATACGACT (positions 2258–2278 on CVA6 Kyoto5 D3 strain, GenBank accession number AB779618.1) and CVA6D3_VP1Ar GGTATGATTTYCTGCTATCCGG (2935–2914), generating a product size of 678bp; and set B: CVA6D3_VP1Bf GCGCTTTGAYGCCGAGTT (2814–2831) and CVA6D3_VP1Br TCTCGTGAGCTACTTTCCCA (3473–3454), generating a product size of 660 bp. Both products were amplified in 20 µL reactions with HotStarTaq (QIAGEN, Hilden, Germany), as per the manufacturer’s recommendations, 1–2 µL of hexamer-primed cDNA template and 200 µM of forward and reverse primers. Then, thermo-cycled as follows: 95 °C for 15 min then 55 cycles of 95 °C for 20 s, 55 °C for 20 s and 72 °C for 60 s. PCR products of the expected size when visualised by gel electrophoresis were sequenced with the primers CVA6D3_VP1Ar and CVA6D3_VP1Bf as appropriate. The sequences were basecalled using FinchTV and identity was preliminarily assessed using NCBI Standard Nucleotide BLAST (BLASTn) and the Genome Detective online Enterovirus Genotyping Tool (Version 2.9) [42].

The sequences were compared to a global reference dataset generated by retrieving all available full length VP1 lineage D sequences CVA6 (*n* = 4358) from GenBank in December 2023, as well as a smaller cohort to identify lineage [35]. These were aligned with the study sequences using Geneious Prime software (Version 2024.0), and a phylogenetic tree was assembled in IQ tree 2 (Version 2.3.6) [43] using the SYM+I+R6 model and 1000 SH-aLRT bootstraps, then annotated using FigTree (Version 1.4.4). The complete CVA6 sequences from this study are deposited on GenBank under accession numbers MZ576353–MZ576381 and PQ611388 –PQ611452.

### 2.3. CVA6 Clinical Audit and Statistical Analysis

The available clinical data were audited under local study number 23-005C, as approved by the Nottingham University Hospitals NHS Trust. As part of the anonymisation process for clinical data export, ages were only recorded in discrete months or years. Without formal prospective categorisation, rash type reporting was highly varied from multiple clinical sources and degrees of experience, including the following: Vesicular (*n* = 30), eczema coxsackium (*n* = 18), HFMD (*n* = 8), crusted lesions (*n* = 2), impetigo (*n* = 2), as well as macular, maculopapular, nappy rash, papular and combinations of the aforementioned types or types not stated (*n* = 16). Therefore, this data category was reduced to the binary presence or absence of a rash for statistical analysis, and accordingly no distinction could be made between classical and atypical HFMD.

Swabs taken from any external anatomy were designated as skin swabs and those involving any sampling of the throat, e.g., nose and throat, as oral swabs. Any non-skin or oral cavity specimen, including whole blood, faeces, nasopharyngeal aspirates (all *n* = 2) and ocular swabs, bronchoalveolar lavage and sputum (all *n* = 1) were grouped together as ‘other’ for the analyses.

Statistical analyses were performed on the available clinical data segregated by the time of sample acquisition. The samples from 2018 were compared against those sampled in 2021–2023. Contingency analyses of the categorical data were performed by χ^2^ test, as implemented in GraphPad Prism 10.3.1.

## 3. Results

### 3.1. Epidemiology

CVA6 was identified in 33 of 143 cases (23.1%) of sequence-confirmed enteroviral diagnoses occurring between 1 September and 17 December 2018, the second most prevalent type behind EV-D68 as we previously reported [39]. The second study period commenced on 11 May 2021 and continued to 26 April 2023, and indicated CVA6 infection in 69 of 185 successfully typed cases (37.3%, the most common in this period). A total of 19 of the study-typed CVA6 cases were independently referred for typing at the National Enterovirus Reference Laboratory and all returned a CVA6 result. Both the study-identified EV-D68 and CVA6 co-infections were also confirmed by the reference lab; however, 79 of the 102 total CVA6-positive cases (77.45%) were not referred for typing.

**Figure 1:**
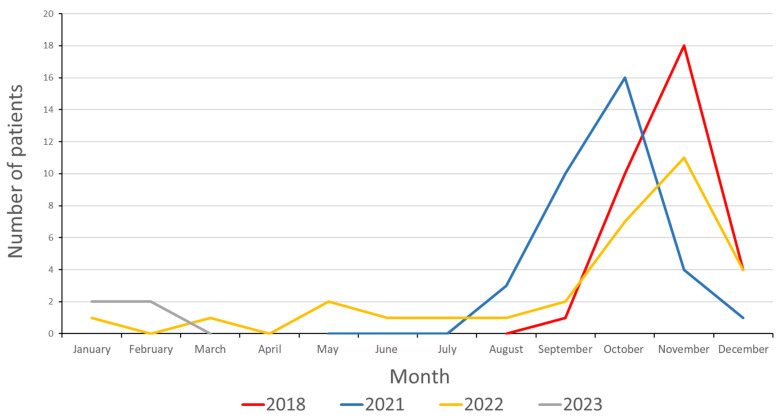
Sequence-confirmed CVA6 prevalence by month. The 2018 samples (red line) were only assessed between September and December, then enteroviral typing recommenced in May of 2021 (blue) and was continued through the entirety of 2022 (yellow) to the end of April in 2023 (grey).

CVA6 infections were seen predominantly between August and December, but in the sole fully screened year of 2022, sporadic incidences were observed throughout. The CVA6 epidemic season of 2018 peaked in November; whereas the first post-pandemic season sampled in 2021 saw an earlier peak in October, followed by a return to a November peak in 2022. The seasonal peak and breadth were also broadly similar in each year, particularly between 2018 and 2021, peaking at 18 and 16 cases per month, respectively, with more cases in the month preceding the peak than in the month after (Figure 1). The overall respiratory illness testing information is presented in Figure S1, indicating a pre-pandemic peak in November of 2018 coinciding with CVA6 epidemiology. Post-pandemic enteroviral testing was at a more consistent but lower level between September and December.

### 3.2. Clinical and Laboratory Characteristics

CVA6 positive patients’ clinical presentations were audited retrospectively, comparing cases recorded before and after the emergence of SARS-CoV-2 (Table 1). The retrospective clinical audit was unable to accurately distinguish between rash presentation and according classical and atypical HFMD presentation based on non-uniform reporting, so these varied presentations were grouped together (see Section 2).

Strikingly, CVA6 presented much more frequently in the clinical audit with a principal diagnosis of HFMD post-pandemic (*p* = 0.0057, Table 1). This was further supported by the increased frequency of a symptomatic rash post-pandemic, noted in 89.9% of cases (*p* = 0.0106, Table 1), whereas the higher level of respiratory involvement pre-pandemic (45.5% compared to 18.8% post-pandemic, *p* = 0.073) tallied with the accordingly lower principal HFMD diagnosis (Table 1). Indeed, 7 of 11 of the non-HFMD primary diagnoses were classified as respiratory and two more involved EV-D68 co-infection.

The overwhelmingly predominant age group presenting with the illness was the young (84 of 102 cases, 82.35%, Table 1 and Figure S2), but not neonatal, with just a single case under 6 months of age, whilst 12 adults were also diagnosed. Alongside the principal differences in CVA6 presentation, the co-morbidities were also significantly elevated post-pandemic (*p* = 0.0018, Table 1). However, this is likely to be strongly linked to the greater proportion of primary HFMD diagnoses, with 20 of 29 post-pandemic instances of co-morbidities noting eczema, a key factor for the exacerbation of HFMD, all but one of which was indeed recorded principally as an HFMD presentation.

Neurological symptoms were also highlighted to be significantly more prevalent pre-pandemic, but only four patients presented as such, one of which presented with an EV-D68 co-infection diagnosed with Acute Flaccid Paralysis (AFP) [39]. Other cases were in young children suffering from febrile convulsions, with one requiring intensive care, while the other did not despite an episode of respiratory arrest. The final neurological case was less severe in a young adult experiencing parathesis in addition to a more typical CVA6 picture of respiratory symptoms, with chest pain, shortness of breath and fever. Additional unusual presentations included single instances of sudden infant death syndrome (SIDS) and conjunctivitis.

In general, the cohort indicated an imbalance favouring the male gender with near identical ratios of approximately six males to four females in both pandemic eras (Table 1). Similarly, the proportions of hospital admissions, fever and gastrointestinal symptoms were also not significantly different pre- and post-pandemic (Table 1). Collectively, these indicated more than half of the cases were assessed as outpatients, and a fever was only reported in around a third of the patients, whilst gastrointestinal symptoms were even less frequent, circa 1 in 10 cases (Table 1). Co-infection by other routinely screened-for viruses [44] was rare and thus not further assessed.

### 3.3. Phylogenetic Analysis of CVA6 VP1 Sequences

To interrogate the genetic relationship between the CVA6 viruses causing infections during our study period, we conducted a phylogenetic analysis of the complete VP1 sequence from both our study samples and the complete available global dataset as of December 2023. The PCR amplification strategy generated a full length VP1 sequence for all but 6 samples (92 of 98 CVA6 infections determined locally, with 4 determined exclusively by the National Enteroviral Reference Laboratory).

As expected, due to the preliminary investigation and final amplification strategy (see Section 2, all samples were of lineage D3 (Figure S3)). Accordingly, only lineage D samples were included in the full phylogenetic tree. However, within this lineage there was considerable diversity not only amongst the reference sequences but also within our local contemporary sequences.

**Figure 2:**
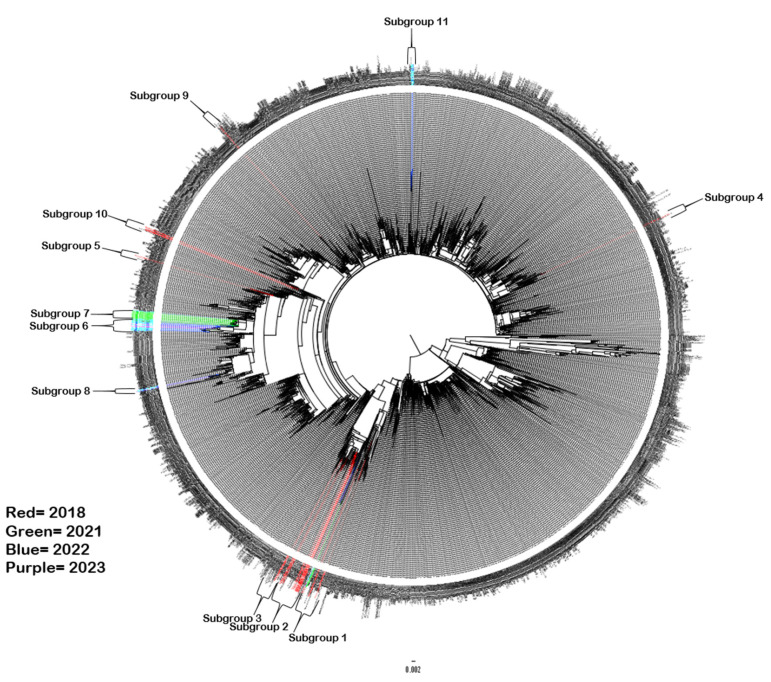
Molecular phylogenetic analysis of complete CVA6 VP1 gene of study and available reference sequences. The phylogenetic tree includes 27 novel sequences from 2018 (red), 28 novel sequences from 2021 (green), 31 novel sequences from 2022 (blue), 5 novel sequences from 2023 (purple) and 4358 publicly available lineage D sequences from GenBank. The tree was constructed using the maximum likelihood method with the SYM+I+R6 model in IQ-TREE2. Bootstrapping based on 1000 replications was performed, bootstrap values were omitted for clarity. Tree is midpoint rooted, with a scale of 0.002. A simplified phylogeny of the study sequences and down-sampled reference set is presented in Figure S4.

**Figure 3:**
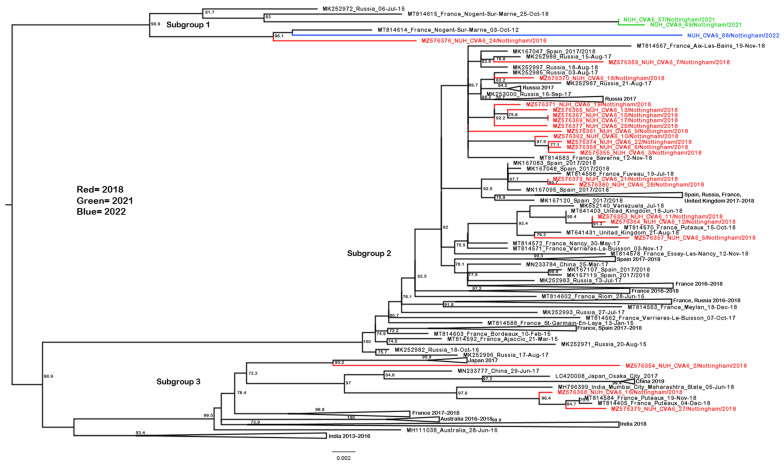
Phylogenetic analysis of CVA6 complete VP1 subgroups 1–3. Molecular phylogenetic analysis of full VP1 CVA6 gene using the maximum likelihood method with the SYM+I+R6 model in IQ-TREE2. Analysis was completed to include the subgroup 1, 2 and 3 sequences highlighted in Figure 2, including 21 novel sequences from 2018 (red), 2 novel sequences from 2021 (green), a novel sequence from 2022 (blue) and 133 publicly available sequences from GenBank. Where branches have been collapsed for clarity (represented by triangles), the country of origin and year for all sequences has been annotated. Bootstrapping based on 1000 replications is shown next to each branch at a scale of 0.002, with bootstrap values below 70 omitted. Tree is midpoint rooted.

The study sequences distributed across genotype D3, were located in 11 different well-supported subgroups (Figure 2 and simplified dataset available as Figure S4), with the pre-pandemic 2018 sequences found exclusively in subgroups 1–5 and 9–10. The majority of these 2018 season sequences (21 of 29) are presented in Figure 3, alongside contemporary 2017–2018 European isolates from elsewhere in the U.K. [45], France and Spain [13], as well as Russia, China and Venezuela. The U.K. study sequences were not always discretely grouped with each other, with some cases showing a closer genetic relationship with geographically distant samples than with each other, e.g., NUH_CVA6_18 presents in a well-supported branch with exclusively Russian sequences (Figure 3, subgroup 2). Subgroup 1 contains only a single 2018 study sequence, but also those from October 2021 and November 2022, alongside the contemporary French and Russian samples (Figure 3). Similarly, subgroup 3 presents an isolated 2018 study sequence (NUH_CVA6_2) on a well-supported branch with the exclusively Japanese sequences from the year before, as well as two sequences (NUH_CVA6_16 and 27) which group amongst the isolates from France and India [46]. Subgroup 4 (Figure S5) contains only a single 2018 study sequence on a long branch in a well-supported grouping with a 2016 Australian sample [47].

Whilst these isolated study sequences are suggestive of intercontinental transmission but lack supporting epidemiological evidence, sample NUH_CVA6_14 (subgroup 5, Figure S6) unusually appeared on a relatively long single branch and presented with a recent travel history to several Southeast Asian countries, not including China. The sequences from this sample comprehensively outgroup the study’s sample. Surprisingly, this patient was not only co-infected with an Enterovirus D68 strain, but this also stood distinct from the contemporary U.K. EV-D68 genome sequences, as we have previously reported [39].

**Figure 4:**
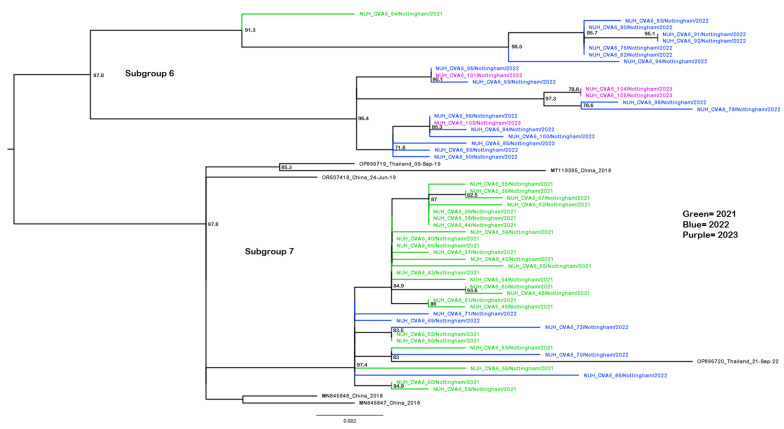
Phylogenetic analysis of CVA6 complete VP1 subgroups 6 and 7. Molecular phylogenetic analysis of full VP1 CVA6 gene using the maximum likelihood method with the SYM+I+R6 model in IQ-TREE2. Analysis was completed to include the subgroup 6 and 7 sequences highlighted in Figure 2, including 26 novel sequences from 2021 (green), 22 novel sequences from 2022 (blue), 4 novel sequences from 2023 (purple) and 6 publicly available sequences from GenBank. Bootstrapping based on 1000 replications is shown next to each branch at a scale of 0.002, with bootstrap values below 70 omitted. Tree is midpoint rooted.

The post-SARS-CoV-2 emergence years and peak seasons of 2021 and 2022 exhibited similar patterns of diversity, with the 2021 sequences predominating in subgroup 7 and the 2022 sequences predominating in subgroup 6 (Figure 4). Each subgroup contained a vice-versa minority, i.e., five of the 2022 sequences amongst the twenty-five 2021 sequences (subgroup 7) and a sole 2021 sequence amongst the seventeen 2022 sequences (subgroup 6). This may be suggestive of local endemic circulation and persistence of the CVA6 strains across the core epidemic seasons as well as lineage displacement (August to December, Figure 1). Subgroup 6 further persisted into 2023 with 4 of the 5 infections investigated that year, albeit before the main epidemic season after the study’s end. In contrast, the dwindling subgroup 7 was not detected. Interestingly, subgroups 6 and 7 were homogenously represented in our U.K. study sequences and out grouped by the well-supported nodes from the 2018 Chinese strains, as were the sparser study sequences in subgroup 8 (Figure S7) in contrast to the aforementioned interspersal of reference sequences seen in subgroup 2 that contained the bulk of the 2018 infections. The smaller 2022 infection-sequenced subgroup 11 was similarly homogenous, but out grouped by a pre-pandemic French isolate (Figure S8) as was an isolated 2018 sequence in subgroup 9 (Figure S9).

Identical sequences were observed in all cases of serially sampled patients, for example, study samples 91 and 92, as well as 104 and 105 (Figure 4). Identical sequences were also derived from different cases, for example, samples 1, 4 and 8 (subgroup 10, Figure S10) and 15 and 17 (Figure 3). However, the clinical audit indicated these were unrelated by family or immediate U.K. Postcode-level geography and thus were unlikely transmitted by direct contact.

## 4. Discussion

Our study covered two sampling periods, both before and after the emergence of SARS-CoV-2, where we undertook universal in-house enteroviral typing, rather than selective referral to a reference laboratory service [30,39]. Whilst a key objective in the pre-pandemic era was to assess the impact of EV-D68 in a hospital setting [39,48], it became apparent that CVA6 was the second-most prevalent enterovirus detected and thus demanded further investigation. This finding was re-enforced when we undertook a post-pandemic pilot project to determine the genotype of all diagnosed infections. The high prevalence of CVA6 amongst the clinically diagnosed enteroviral infections was in agreement with other universal screening studies, most significantly and relevantly in the recent and largest study of enteroviral infections in Europe to date [4] but also in the U.S. [49] and worldwide in general [40].

The seasonality of enterovirus infections can sometimes lead to them being oversimplified as broadly summer infections [50], which may be true for some types but not others [4]. However, here we demonstrate that, in agreement with others, CVA6 undoubtedly peaks in the Autumn in the U.K. [51]. A subtle shift in the transmission peak was observed in 2021, potentially due to the unprecedented non-pharmaceutical intervention measures introduced to limit SARS-CoV-2 transmission in the U.K. and elsewhere globally [52]. Diminished post-pandemic transmission rates for other enteroviruses have also been recorded [48] with an accompanying uncertainty about the infection rates and severity as interventional measures were lifted within a generally older, susceptible population [53].

Age at CVA6 diagnosis broadly followed trends reported elsewhere, with a large U.K.-based cohort also finding most patients (52%) to be 1–5 years of age and a further quarter (23%) to be between 4 and 12 months old [51], as well as a similar demographic trend seen more broadly in Europe [4]. Interestingly, our hypothesized but not statistically significant study trend of children acquiring CVA6 slightly later in life in the immediate post-pandemic era was in contradiction to a similar study performed in the Netherlands [38].

By comparison, 2021 Respiratory Syncytial Virus infections were recorded earlier than expected and also with increased severity [54,55]. The perturbance of typical viral transmission patterns has been assessed most intensively with regards to influenza epidemiology [56] and observed most significantly in the lack of detection of Influenza B Yamagata lineage since March 2020 [57]. Although influenza epidemics were observed to be of lower intensity and shorter in a study of nine Asian countries in 2021 [58], our data indicated a similar size CVA6 infection burden in 2021 compared to 2018. The greater proportion of males presenting at circa 60% in each group appears to be a general feature of HFMD as this was in close agreement with a predominantly EV-A71- and CVA16-causal HFMD cohort in China [59].

Whilst our limited CVA6 cohort split across three epidemic seasons with non-uniformly recorded clinical details was unable to fully probe disease manifestations, particularly in terms of rash appearance, others have done so comprehensively in contemporary European pre-pandemic cohorts, finding an approximately 3 to 1 caseload of atypical rather than typical HFMD presentation [37]. Instead, we focused on the more universal parameters of infection and their variability pre- and post-pandemic, finding elevated respiratory tract involvement in 2018, supported by the findings in linked parameters with more respiratory tract sampling and lesser rash observation and the HFMD primary diagnoses in this year. The pre-pandemic sample profile aligns more closely with the results from the broad European cohort of Bubba and colleagues, with CVA6 detected by skin swab in approximately half of all cases and secondarily in the respiratory tract in approximately a quarter of cases [4]. Others have found CVA6 to be the most commonly detected enterovirus of the respiratory tract [60]. In another extensive, but more closely matched study set exclusively in the U.K. from 2006 to 2017 (>1000 CVA6 cases), the sample set comprised 73.5% skin (and vesicle) swabs and just 13.2% respiratory specimens, similar to our more disparate post-pandemic sampling [51].

The limited sampling from non-skin/oral sites and the associated non-respiratory/HFMD principal diagnosis seen in our results, was reflected elsewhere [4,51], although the circa one-third of febrile cases recorded in both the pre- and post-pandemic groups was approximately three-fold greater than the European average [4]. However, neurological manifestations such as the febrile convulsions we saw in the smaller 2018 group have been significant proportions of other CVA6 cohorts [60]. The severe neurological complication AFP was also seen in CVA6 cases in China earlier in the mid-2000s [35], although our sole case was co-infected with EV-D68, which may have been the principal causative agent [39,61]. EV-D68 and CVA6 co-infection has also been observed previously, presenting classical symptomology for both enteroviruses [62]. Given that these viruses were the most prevalent in our recent surveillance pre- and post-pandemic here and elsewhere [39], occasional co-infections should accordingly be expected, as should co-infection by the intra-typic strains of CVA6 that must have facilitated the extensive genomic recombination characterised in whole genome sequencing studies [27,28,37].

We confirmed the recent dominance of the CVA6 genotype D3 in the U.K., as was reported both elsewhere in continental Europe [13,37] and China [35], although our exclusively VP1 sequencing approach lacked the ability to further characterise the aforementioned genomic recombinants hypothesised to enhance its post-millennium emergence and pathogenicity [27,37,63]. However, whole genome sequencing only further exacerbates the apparent complexity of the strains beyond the genotype D3 designation that was apparent in our phylogenetic analyses. Whilst we observed less intermingling between study and non-study sequences post-pandemic, this is likely to be at least in part an artefactual result of the limited available reference material to date as many viral sequencing laboratories have diverted time and resources to prioritise SARS-CoV-2 studies.

In the better-sampled pre-pandemic era, considerable sub-genotype diversity and by implication labyrinthine transmission chains were indicated. Closely sampled CVA6 genomes from our regional U.K. centre were more closely related to those in other countries or even continents than to each other in many instances, as was also the case in our related EV-D68 study [39]. The intercontinental viral transmission illustrated by groups such as ours during the emergence of SARS-CoV-2 [64] was again demonstrated with the likely import of an East Asian CVA6 sub-lineage alongside an East Asian EV-D68 sub-lineage [39], most surprisingly in the same co-infected returning traveller. Where the pre-pandemic study sequences were most closely related, or even identical in the VP1 region covered, clinical auditing indicated no obvious clues as to the epidemiological linkage, further emphasising the hidden subclinical transmission events and the unsampled burden of infection. A study sampled in the preceding years of 2016–2017 in Australia also showed a similarly mixed epidemiological picture of genetically isolated viruses alongside clusters suggestive of endemic transmission chains across seasons [47].

Others have attempted to compare enteroviral genetic diversity pre- and post-pandemic, finding a reduction in genetic variability may have occurred with the lockdown measures introduced, although not notably in CVA6 [38]. Whilst suitably cautious in waiting for additional evidence from further complementary studies such as the one presented here, they equally note that genetic change arising from transmission bottlenecks may indeed become clinically relevant in the future.

We also remain tentative in our assertion of the presented results and their interpretation as our study experienced several limitations. We reiterate the non-uniformity of the clinical data available from a retrospective audit rather than a prospective purpose-designed cohort. This was compounded by a single pre-pandemic CVA6 epidemic season of only four months, followed by an approximately 30-month gap before we resumed our intensive enteroviral focus. We further reemphasise that single gene sequencing overlooks the additional genetic complexity better characterised by the increasingly accessible whole genome approach.

In conclusion, the global epidemiology of viral infections is experiencing an unprecedented era, following a phase of restrictive measures and their subsequent relaxation, leaving an atypical global immune landscape within which to renew transmission [65]. Our study indicates this phenomenon has indeed perturbed CVA6 in the U.K., with evidence of an effect on circulating lineages, their peak epidemic season and their associated clinical manifestation. The global capability in virological surveillance demonstrated during the COVID-19 pandemic could be increasingly applied to monitor the ever-changing epidemiology of enteroviral infections [66].

## Figures and Tables

**Table 1:** Clinical and laboratory data from the 102 CVA6-positive study cases.

	Pandemic Era		
	Pre-	Post-	
**Cases**	33	69	
**Clinical Feature**	N (%)	N (%)	*p* Value
Male gender	20 (60.6)	43 (62.3)	0.866
Hospital admission	14 (42.4)	24 (34.8)	0.4552
Comorbidities	3 (9.1)	26 (37.7)	0.0018
Neurological symptoms	4 (12.1)	0 (0.0)	0.0039
Fever	13 (39.4)	21 (30.4)	0.4543
Gastro	4 (12.1)	7 (10.1)	0.8211
Rash	23 (69.7)	62 (89.9)	0.0106
Respiratory symptoms	15 (45.5)	13 (18.8)	0.073
HFMD primary diagnosis	22 (66.7)	59 (85.5)	0.0057
**Sample site**			
Skin swab	15 (45.5)	59 (85.5)	<0.0001
Oral swab	13 (39.4)	6 (8.7)	
Other sample	5 (15.2)	4 (5.8)	
**Age group**			0.5569
Under 1 year	9 (27.3)	13 (18.8)	
1 year	15 (45.5)	27 (39.1)	
2 years	3 (9.1)	11 (15.9)	
3 years	0 (0.0)	6 (8.7)	
4 to 17 years of age	1 (3.0)	5 (7.2)	
Adult	5 (15.2)	7 (10.1)	

Patients were grouped into pre- (1 September to 17 December 2018, *n* = 33) and post-pandemic (11 May 2021 to 26 April 2023, *n* = 69) cohorts for analysis. Categorical analyses were performed using a χ^2^ test, comparing each clinical feature of the infection, the site of sampling, and age at the time of sampling.

## Data Availability

The original sequencing data presented in this study are openly available in GenBank as described in the Section 2. The additional raw data supporting the conclusions of this article will be made available by the authors upon request.

## Supplementary Materials

The following supporting information can be downloaded at https://www.mdpi.com/article/10.3390/pathogens13111020/s1: Figure S1: Proportion of respiratory diagnostic test results in pre- and post- pandemic eras; Figure S2: CVA6 case proportion by age grouping in pre- and post-pandemic eras; Figure S3: Lineage analysis of CVA6 complete VP1 gene; Figure S4: Simplified molecular phylogenetic analysis of the complete CVA6 VP1 gene of study and available reference sequences; Figure S5: Phylogenetic analysis of CVA6 complete VP1 subgroup 4; Figure S6: Phylogenetic analysis of CVA6 complete VP1 subgroup 5; Figure S7: Phylogenetic analysis of CVA6 complete VP1 subgroup 8; Figure S8: Phylogenetic analysis of CVA6 complete VP1 subgroup 11; Figure S9: Phylogenetic analysis of CVA6 complete VP1 subgroup 9; and Figure S10: Phylogenetic analysis of CVA6 complete VP1 subgroup 10.
