# Supplementary material for: Coxsackievirus A6 U.K. Genetic and Clinical Epidemiology Pre- and Post-SARS-CoV-2 Emergence
Source: Pathogens. 2024 Nov 20;13(11):1020. doi: 10.3390/pathogens13111020 (PMC11597771; doi:10.3390/pathogens13111020)
Supplement: Supplementary file 1 [file pathogens-13-01020-s001.zip › pathogens-3312560-supplementary.pdf]

Supplementary commentary and figures

Additional commentary relating to section 3.3 Phylogenetic analysis of CVA6 VP1 sequences regarding PCR assay failure:

Three samples failed to amplify only with primer set A (bases 2258 - 2935), one with primer set B only (bases 2814 - 3473) and two with both primer sets (but had been confirmed CVA6 by preliminary sequencing, reference [30], main article). Using available sequence data, a third base position mismatch was identified in the reverse priming site of primer set A, however this was also common in successfully amplified samples, suggesting a further undetected mismatch in the forward primer of set A was responsible for PCR failure. Similarly, no mismatches could be identified in the forward priming region of set B, suggesting an isolated mismatch in the reverse set B primer. Two of the A amplicon failures were of low template input, as was one of the two samples that failed for both, whilst the other dual failure was the sputum sample. Partial VP1 sequences were also of common D3 lineage, based on phylogenetic reconstuction using the same reference sequences as Figure S3.

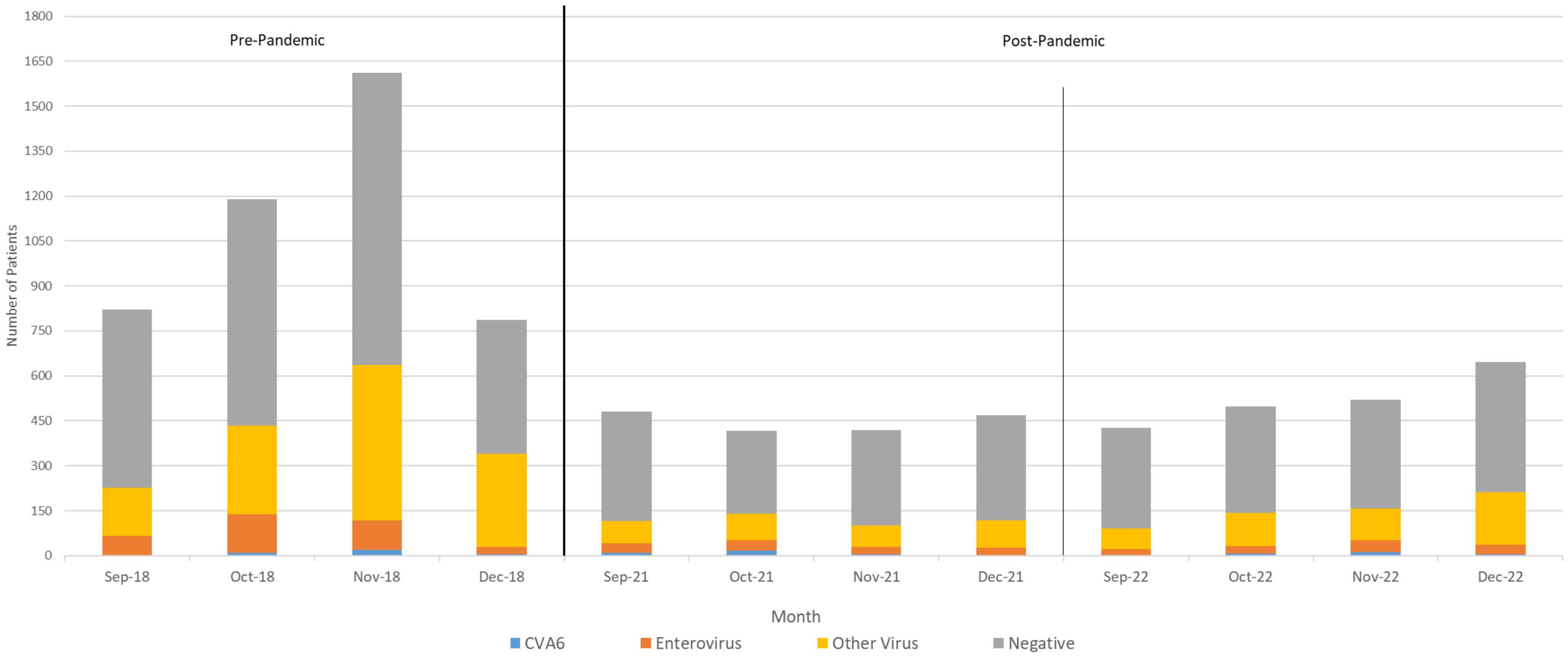

Figure S1. Proportion of respiratory diagnostic test results in pre- and post- pandemic eras. Total number of patients is represented by full length bars. Pre-pandemic era covers 1st September to 17th December 2018, post-pandemic era covers 11th May 2021 and 26th April 2023. Only data for September to December of each year is presented as this encompasses the peak period for CVA6 infection (see Figure 1). Enteroviral testing was generally reduced post-pandemic due to an increased local focus on point-of-care-testing for SARS-CoV-2, Influenza A & B and Respiratory Syncytial virus at admission [reference 52, main article].

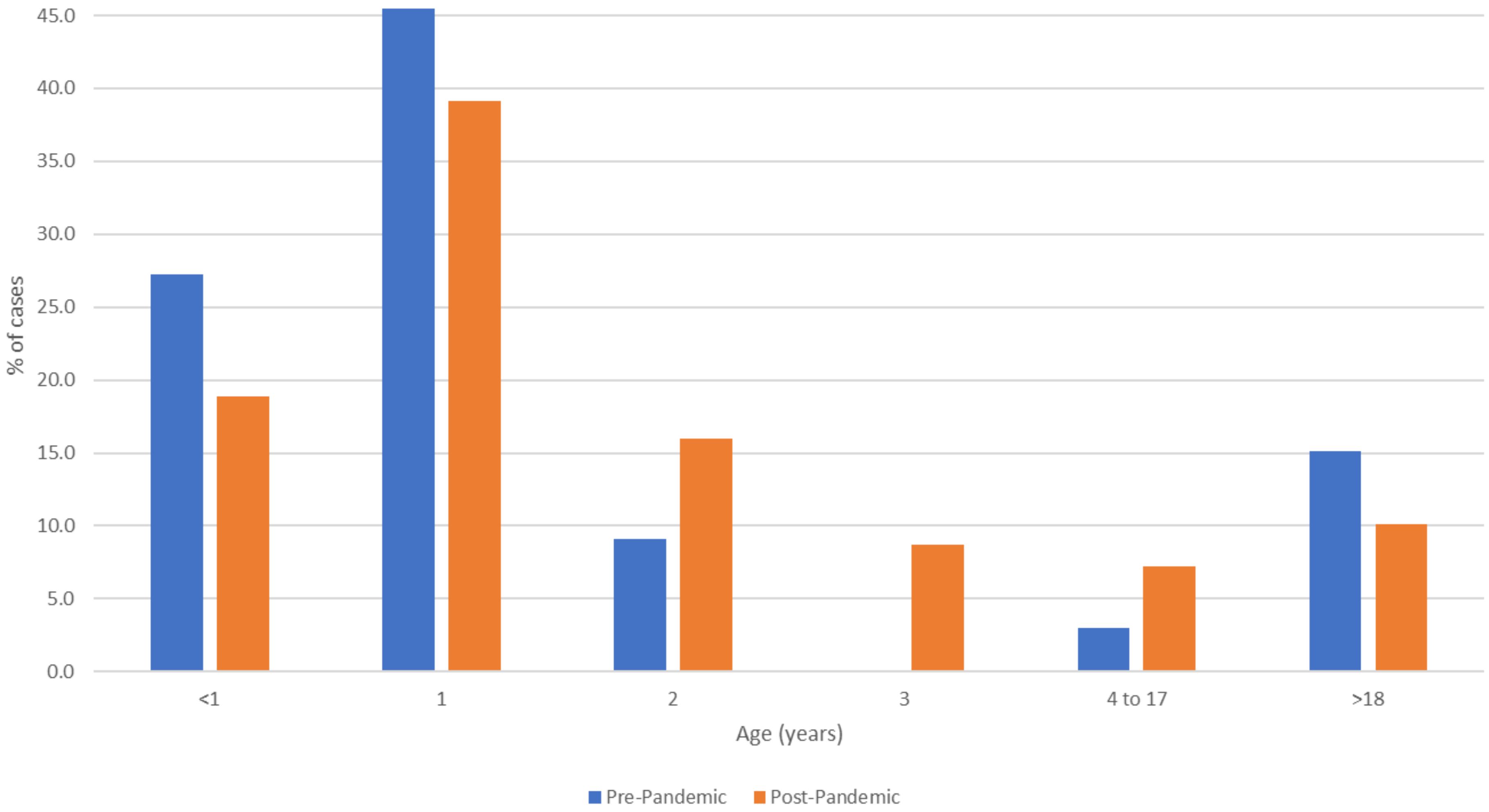

Figure S2. CVA6 case proportion by age grouping in pre- and post-pandemic eras.

Pre-pandemic era covers 1st September to 17th December 2018, post-pandemic era covers 11th May 2021 and 26th April 2023.

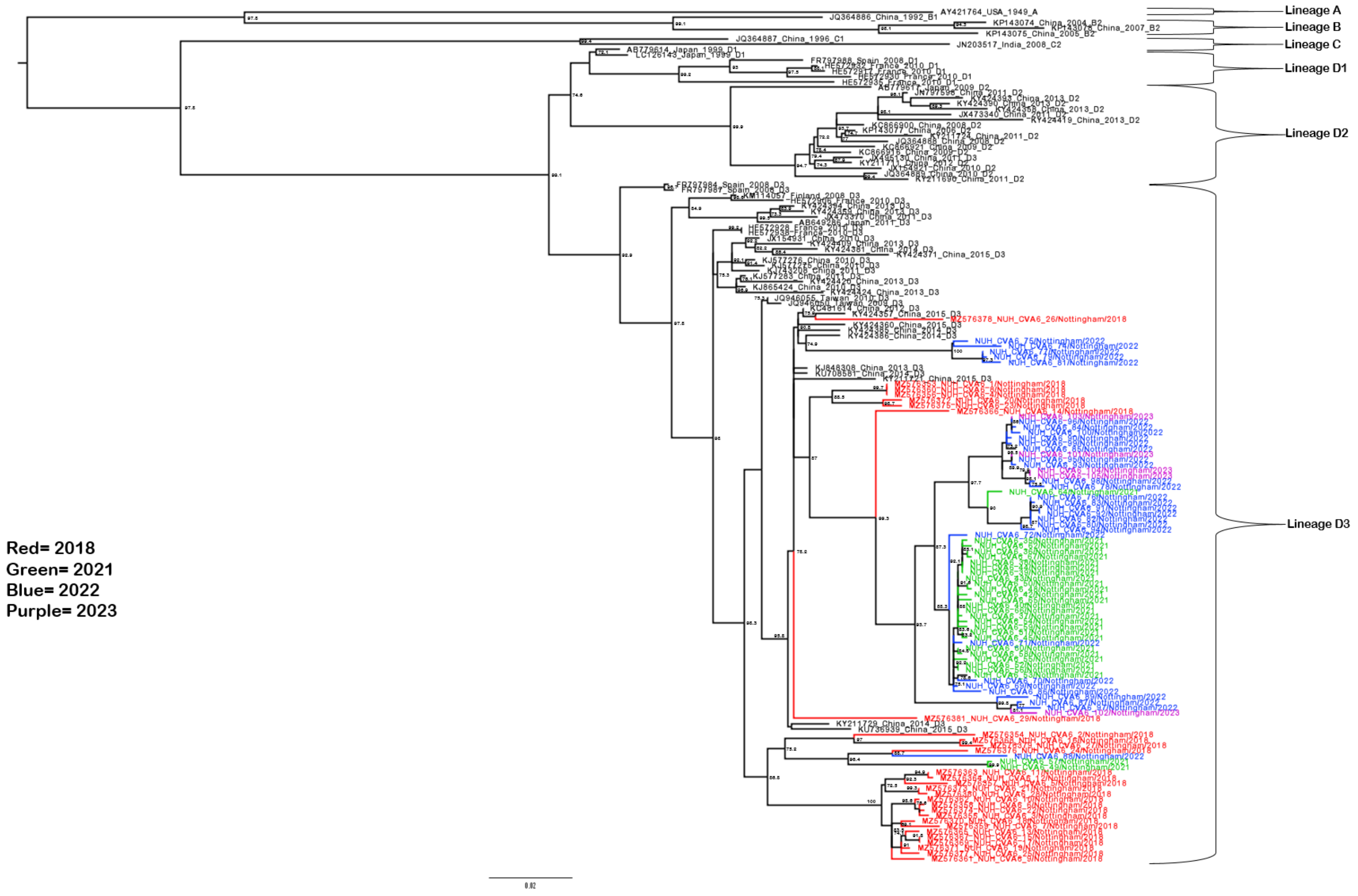

Figure S3. Lineage analysis of CVA6 complete VP1 gene. Molecular phylogenetic analysis of full VP1 CVA6 gene using the maximum likelihood method with SYM+I+G4 model in IQ-TREE2. Includes all novel sequences from this study and illustrates that they cluster amongst lineage D3 sequences. Also includes all lineage A, B, C and D using the dataset from Song et al., 2017 [37], with lineages annotated. Bootstrapping based on 1000 replications is shown next to each branch at a scale of 0.002, with bootstrap values below 70 omitted. Tree is midpoint rooted.

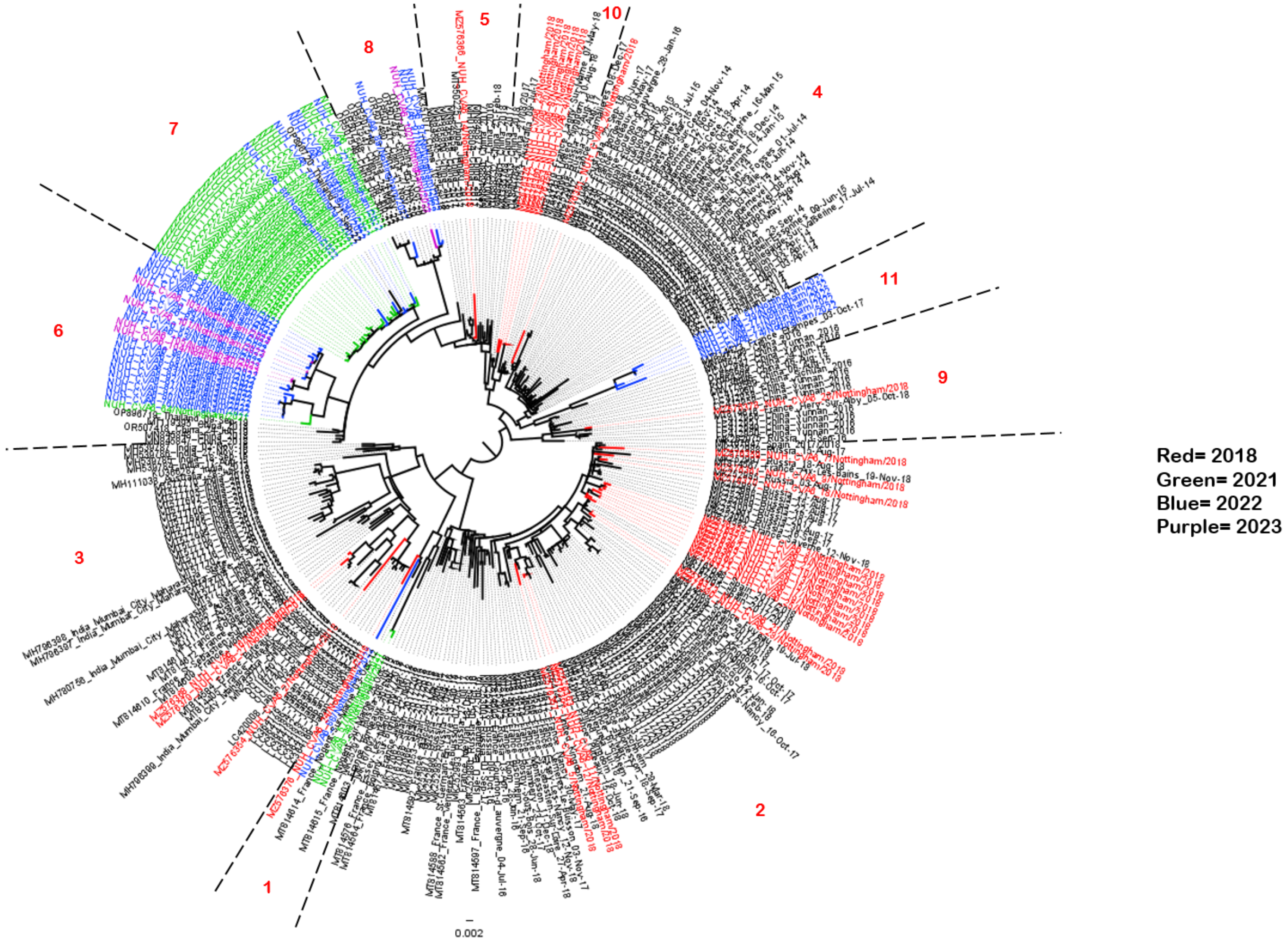
1
2
3
4
5
6
7
8
9
10
11
0.002
Red= 2018
Green= 2021
Blue= 2022
Purple= 2023

Figure S4. Condensed molecular phylogenetic analysis of complete CVA6 VP1 gene of study and down-sampled reference sequences. Molecular phylogenetic analysis of 29 novel sequences from 2018 (red), 28 novel sequences from 2021 (green), 31 novel sequences from 2022 (blue), 5 novel sequences from 2023 (purple) and 222 publicly available lineage D sequences from GenBank. Reference sequences were selected based upon their bootstrap supported segregation into Subgroups 1 to 11 in the complete global CVA6 VP1 phylogenetic tree presented in Figure 2 (main article). Tree was constructed using the maximum likelihood method with SYM+I+R6 model in IQ-TREE2. Bootstrapping based on 1000 replications was performed but bootstrap values were omitted for clarity. Dashed lines represent subgroup divisions, red numbers represent subgroup number. Tree is midpoint rooted, with a scale of 0.002.

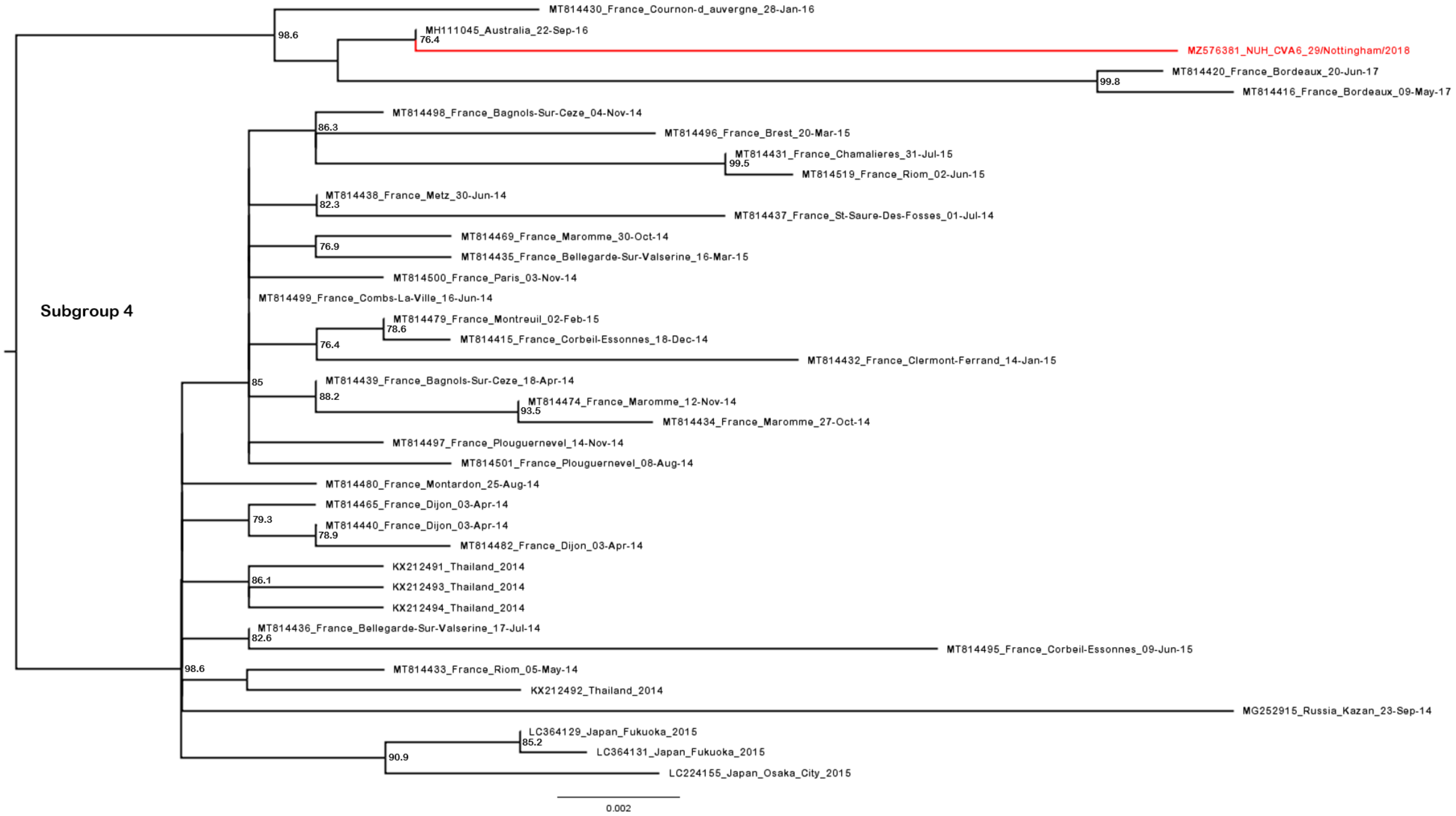

Figure S5. Phylogenetic analysis of CVA6 complete VP1 subgroup 4. Molecular phylogenetic analysis of full VP1 CVA6 gene using the maximum likelihood method with SYM+I+R6 model in IQ-TREE2. Analysis was completed to include the subgroup 4 sequences highlighted in Figure 2 (main article), including a novel sequence from 2018 (red) and 37 publicly available sequences from GenBank. Bootstrapping based on 1000 replications is shown next to each branch at a scale of 0.002, with bootstrap values below 70 omitted. Tree is midpoint rooted.

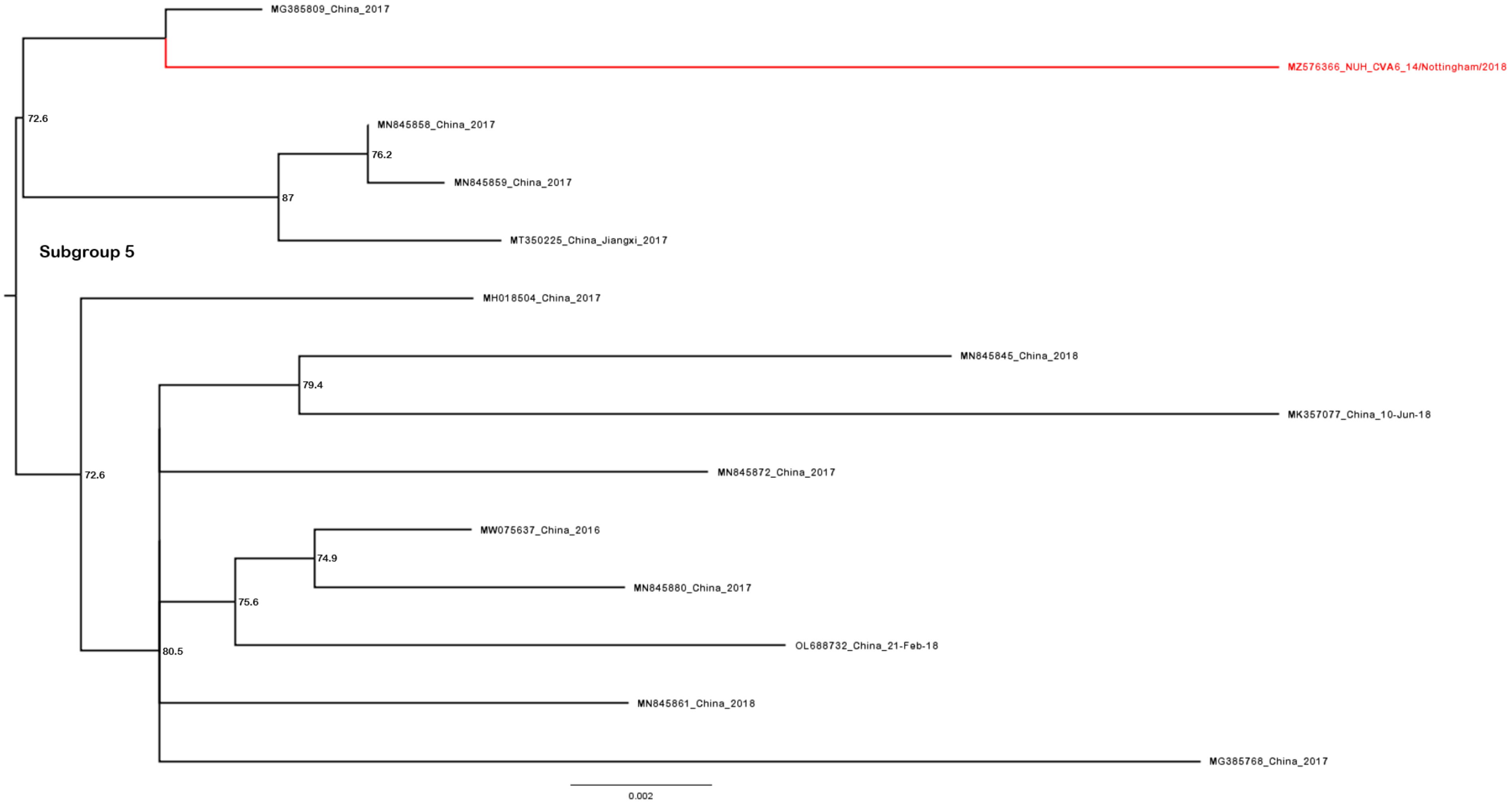

Figure S6. Phylogenetic analysis of CVA6 complete VP1 subgroup 5. Molecular phylogenetic analysis of full VP1 CVA6 gene using the maximum likelihood method with SYM+I+R6 model in IQ-TREE2. Analysis was completed to include the subgroup 5 sequences highlighted in Figure 2 (main article), including 1 novel sequence from 2018 (red) and 13 publicly available sequences from GenBank. Bootstrapping based on 1000 replications is shown next to each branch at a scale of 0.002, with bootstrap values below 70 omitted. Tree is midpoint rooted.

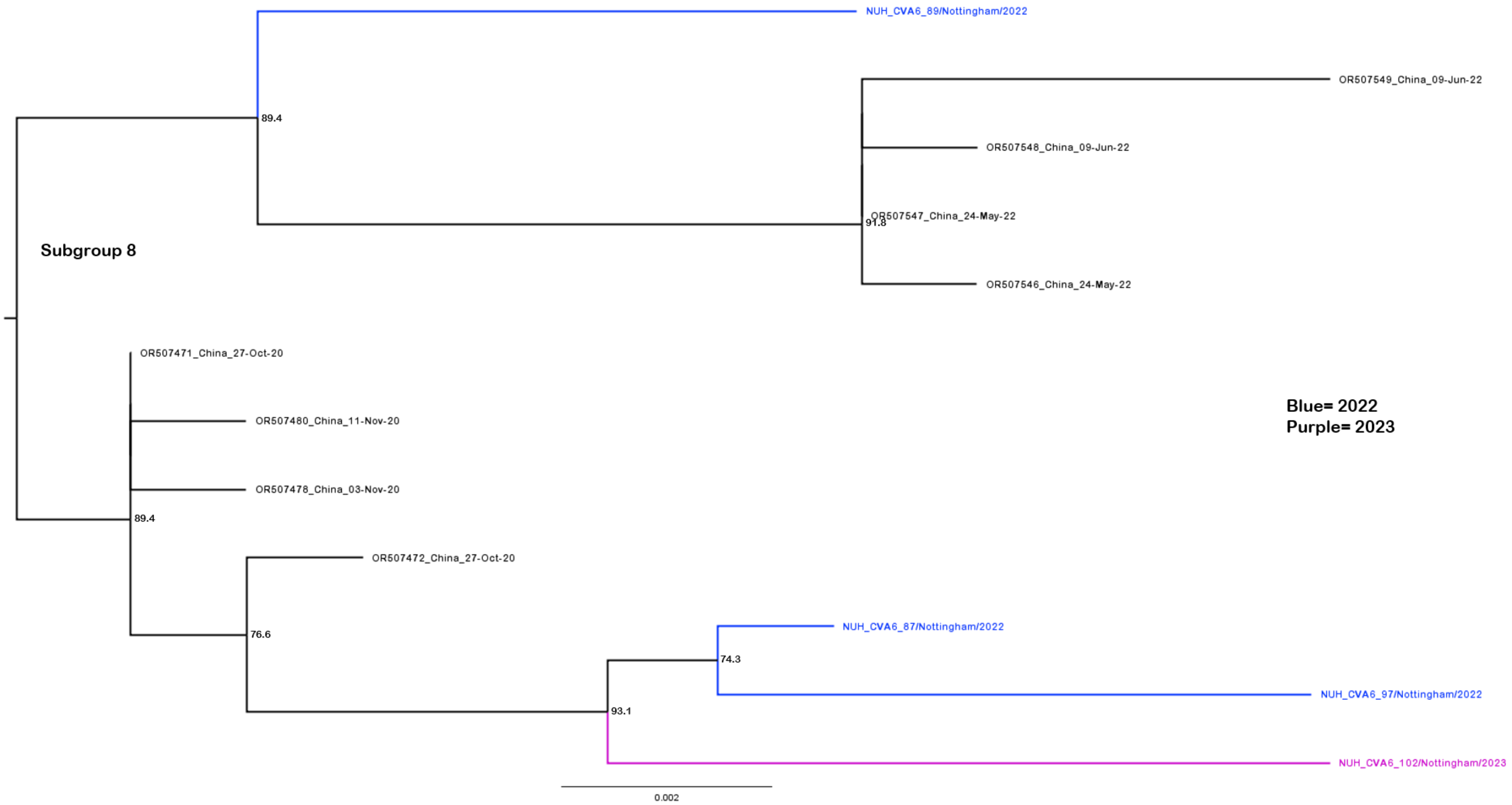

Figure S7. Phylogenetic analysis of CVA6 complete VP1 subgroup 8. Molecular phylogenetic analysis of full VP1 CVA6 gene using the maximum likelihood method with SYM+I+R6 model in IQ-TREE2. Analysis was completed to include the subgroup 8 sequences highlighted in Figure 2 (main article), including 3 novel sequences from 2022 (blue), 1 novel sequence from 2023 (purple), and 8 publicly available sequences from GenBank. Bootstrapping based on 1000 replications is shown next to each branch at a scale of 0.002, with bootstrap values below 70 omitted. Tree is midpoint rooted.

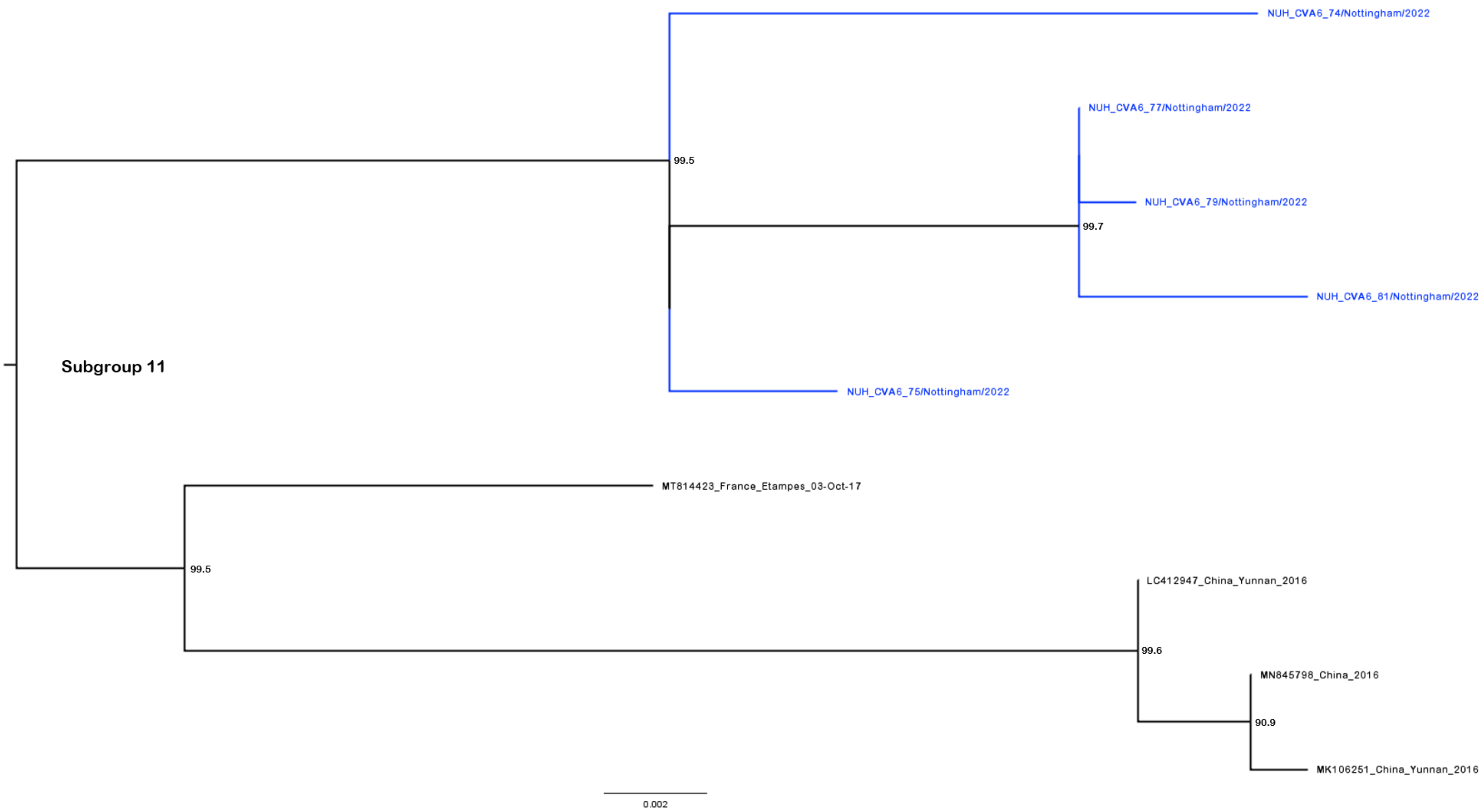

Figure S8. Phylogenetic analysis of CVA6 complete VP1 subgroup 11. Molecular phylogenetic analysis of full VP1 CVA6 gene using the maximum likelihood method with SYM+I+R6 model in IQ-TREE2. Analysis was completed to include the subgroup 11 sequences highlighted in Figure 2 (main article), including 5 novel sequences from 2022 (blue) and 4 publicly available sequences from GenBank. Bootstrapping based on 1000 replications is shown next to each branch at a scale of 0.002, with bootstrap values below 70 omitted. Tree is midpoint rooted.

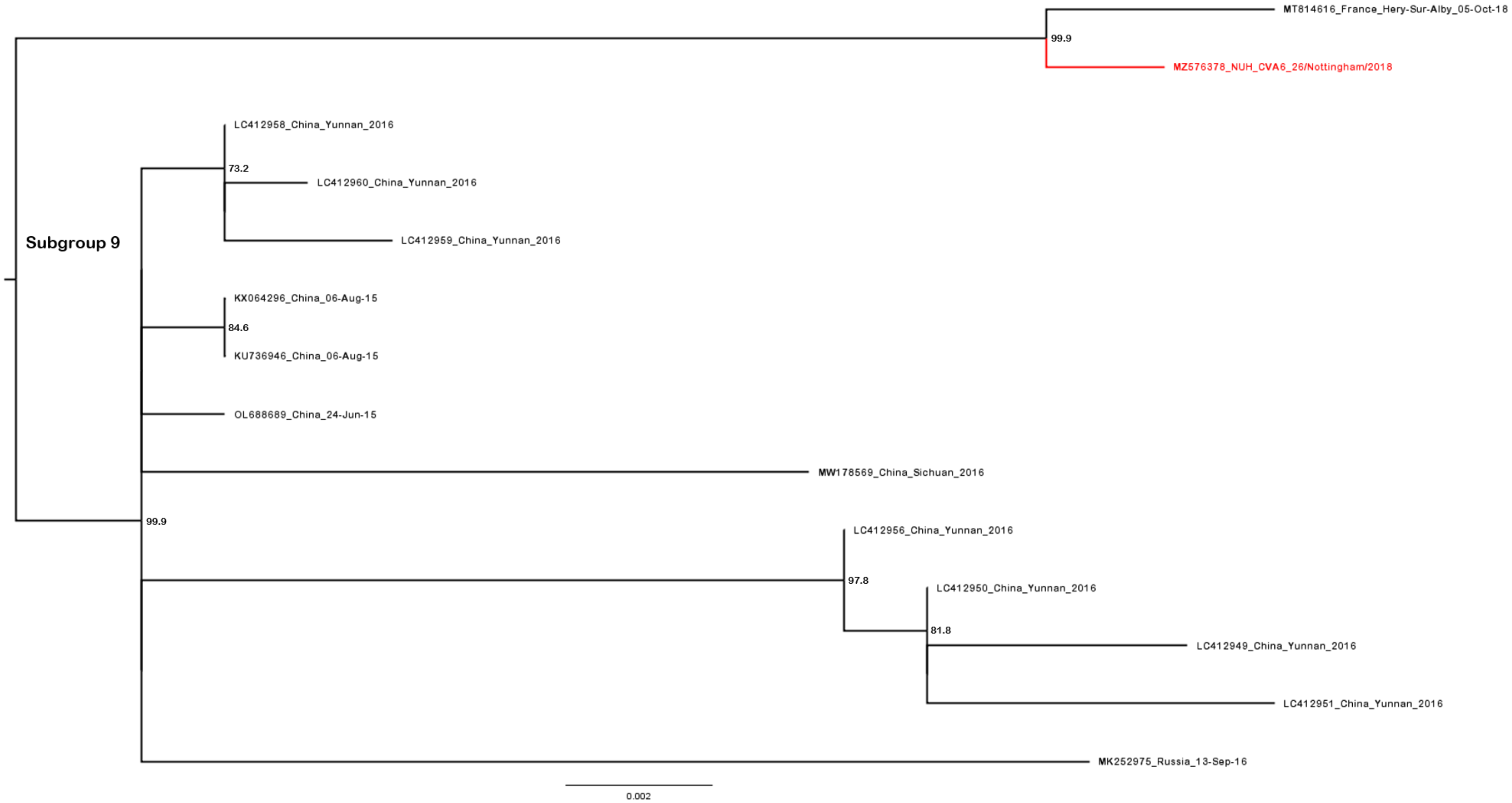

Figure S9. Phylogenetic analysis of CVA6 complete VP1 subgroup 9. Molecular phylogenetic analysis of full VP1 CVA6 gene using the maximum likelihood method with SYM+I+R6 model in IQ-TREE2. Analysis was completed to include the subgroup 9 sequences highlighted in Figure 2 (main article), including 1 novel sequence from 2018 (red) and 13 publicly available sequences from GenBank. Bootstrapping based on 1000 replications is shown next to each branch at a scale of 0.002, with bootstrap values below 70 omitted. Tree is midpoint rooted.

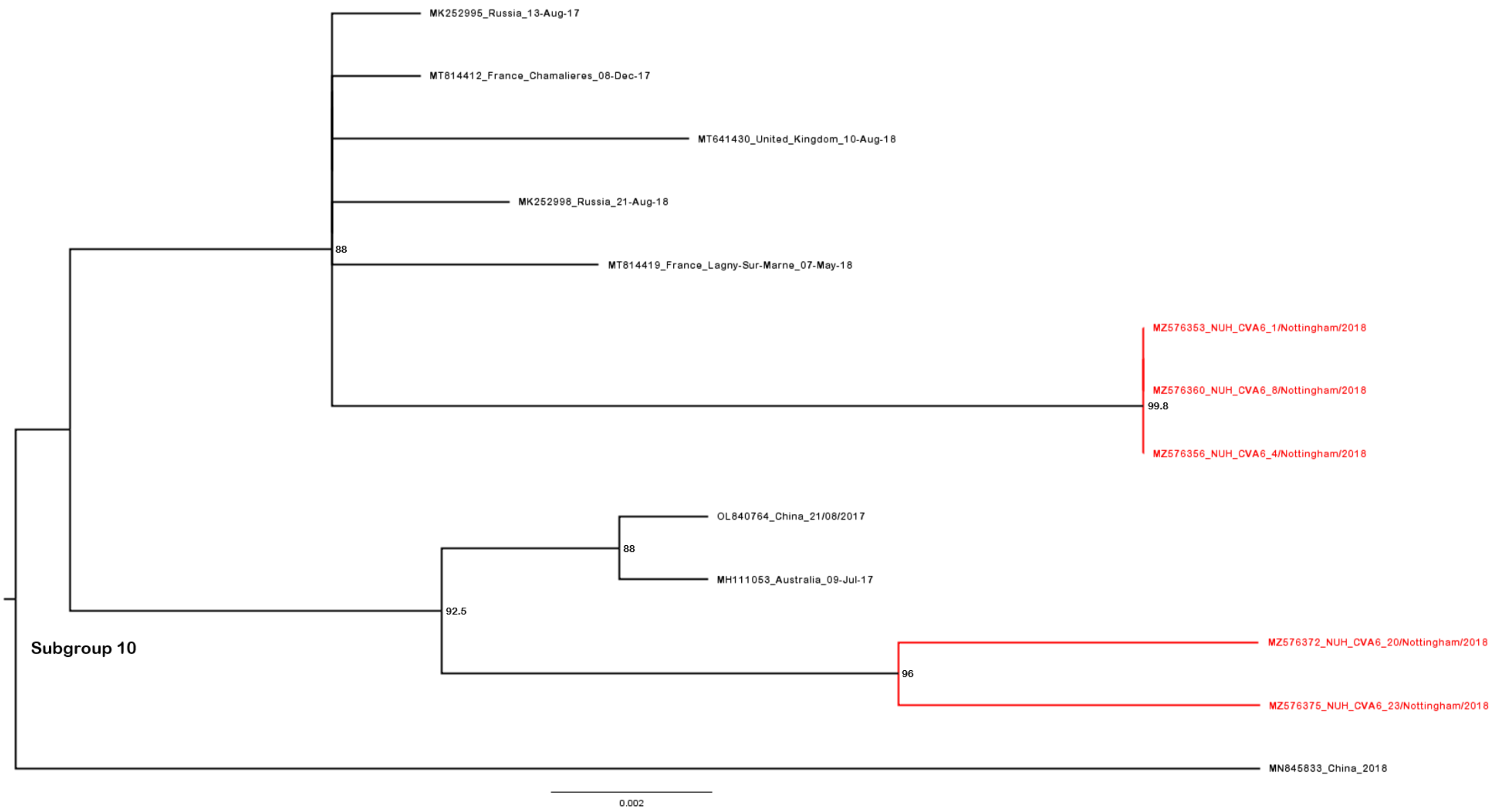

Figure S10. Phylogenetic analysis of CVA6 complete VP1 subgroup 10. Molecular phylogenetic analysis of full VP1 CVA6 gene using the maximum likelihood method with SYM+I+R6 model in IQ-TREE2. Analysis was completed to include the subgroup 10 sequences highlighted in Figure 2 (main article), including 5 novel sequences from 2018 (red) and 8 publicly available sequences from GenBank. Bootstrapping based on 1000 replications is shown next to each branch at a scale of 0.002, with bootstrap values below 70 omitted. Tree is midpoint rooted.
